# Retrieval-Augmented Language Models Enable Scalable Chemical Source Classification in Metabolomics Workflows

**DOI:** 10.1021/acs.analchem.5c05301

**Published:** 2026-01-29

**Authors:** Prajit Rajkumar, Runbang Tang, Harshada Sapre, Jasmine Zemlin, Victoria Deleray, Jeong In Seo, Siddharth Mohan, Shipei Xing, Harsha Gouda, Yasin El Abiead, Shirley M. Tsunoda, Haoqi Nina Zhao, Pieter C. Dorrestein

**Affiliations:** † Collaborative Mass Spectrometry Innovation Center, 8784 University of California San Diego, La Jolla, California 92093, United States; ‡ Skaggs School of Pharmacy and Pharmaceutical Sciences, 8784 University of California San Diego, La Jolla, California 92093, United States; § Department of Civil and Environmental Engineering, Stanford University, Stanford, California 94305, United States; ∥ Center for Microbiome Innovation, University of California San Diego, La Jolla, California 92093, United States

## Abstract

There is a growing need for scalable chemical classification to support the interpretation of exposomics and metabolomics data. While structural categorization has been largely automated, functional and exposure-based labeling of chemicals remains a manual and time-consuming process. Here, we present *chemsource*, a flexible framework that integrates large language models (LLMs) with retrieval-augmented generation (RAG) to automate chemical classification. *chemsource* retrieves descriptive text from Wikipedia or PubMed abstracts based on chemical names and prompts LLMs to assign user-defined categories based on the retrieved content. We demonstrate classification into five exposure categories: endogenous metabolites, food molecules, drugs, personal care products, industrial chemicals, and combinations of these possibilities. Benchmarking against manually curated labels for 4,953 compounds showed 75% overall agreement, with category-level recall exceeding 75% across all classes. Expert review indicated that most discrepancies could be attributed to prompt ambiguity and incomplete manual labels rather than model failure. To demonstrate the utility of *chemsource* in metabolomics workflow, we applied it to eight public untargeted metabolomics data sets, revealing distinct exposure patterns across human biospecimens, mouse tissues, environmental dust, and consumer product extracts. *chemsource* is customizable via prompt editing, enabling diverse classification tasks without requiring coding expertise. The tool is freely available as a Python package (https://pypi.org/project/chemsource/). Text retrieval is free; classification requires user-supplied LLM API access.

## Graphical abstract

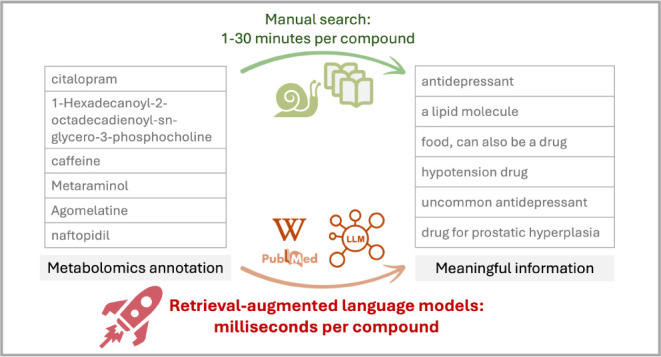

## Introduction

In LC-MS/MS-based untargeted metabolomics, initial compound annotations are typically generated by matching experimental MS/MS spectra against reference libraries of known molecules. The resulting output is a list of candidate compounds, ion forms (e.g., [M+H]+, [M+Na]+), and associated names or structures. To interpret these annotations, researchers often seek additional contextual information, such as a molecule’s biochemical role, biological origin, function, occurrence, or relevance to health and disease. This process usually involves time-consuming web and literature searches, which are increasingly impractical at scale. Additionally, many compounds are known by multiple alternative names that must be considered during searches. As annotation rates have increased ∼10-fold over the past decade and millions of new scientific articles are published annually,^1–3^ comprehensive manual review for every compound is no longer feasible. While expert curation remains valuable, scalable computational assistance is needed to support the biological interpretation of the ever-expanding metabolomics data sets.

Structural classification and pathway mapping are well-established approaches for conceptualizing metabolomics data.^4,5^ However, additional functional or source-based classifications—such as whether a molecule originates from food, functions as a pharmaceutical agent, is used in industrial processes, or is produced by the microbiome—can offer meaningful insights and broaden the scope of questions that metabolomics can address.^4,6–8^ For example, distinguishing endogenous from exogenous metabolites revealed associations between chemical exposure and host metabolic states.^8^ Categorizing exogenous compounds by use categories uncovered exposure patterns that vary with industrialization.^7^ Unlike structural classification, which has been largely systematized through computational algorithms,^9,10^ functional or source-based classification of chemicals remains a manual and labor-intensive process. It typically involves manual parsing of a large amount of scientific literature or encyclopedic resources. Current attempts to automate this process often rely on curated chemical databases,^11–13^ which requires substantial downstream curation to achieve satisfactory accuracy. Additionally, for most classifications, no single authoritative database exists. Instead, the World Wide Web and scientific literature function as a vast but disorganized repository of knowledge, which remains difficult to harness systematically at scale.

The emergence of large language models (LLMs) has gained significant traction in text mining by enabling the direct extraction of semantic information from unstructured text.^14–16^ Although the application of LLMs in biochemistry domains is still in the early stages, recent efforts have demonstrated promising performance in tasks such as summarizing exposure details from complex medical records,^15,17,18^ extracting toxicological profiles of compounds,^19^ retrieving chemical synthesis protocols,^20,21^ and mapping research trends from the literature.^22,23^ Building on this progress, we hypothesize that LLMs can be harnessed to perform user-defined classification of chemicals, offering a scalable and efficient alternative to manual curation. This approach holds the potential to substantially reduce the burden of chemical contextualization in metabolomics workflows by reducing the reliance on labor-intensive literature searches and expert review.

Here, we present *chemsource*, a flexible framework that assists in functional compound classification powered by LLMs. *chemsource* first retrieves textual information about a compound from Wikipedia or the PubMed abstract database, which is then paired with a natural language prompt and submitted to the LLM for classification. The default prompt of *chemsource* is optimized for exposomics applications and categorizes compounds into five exposure-relevant classes: endogenous metabolites, food-derived molecules, pharmaceuticals, personal care products, and industrial chemicals, in which the LLM is instructed to return all categories supported by the retrieved text rather than forcing a single label. The application domain of *chemsource* can be easily expanded: because the model prompt is written in natural language, users can customize the prompt to define alternative categories without programming expertise. We benchmarked *chemsource* using GPT-4o and DeepSeek-V3 ^24^ with different retrieval-augmented generation (RAG) schemes, against expert-curated labels for 4,953 compounds in our newly developed GNPS Drug Library.^25^ *chemsource* is freely available as a Python package on the Python Package Index (PyPI).

## Methods

### Information Retrieval

*chemsource* employs a retrieval-augmented generation (RAG) strategy to enhance the performance of LLMs in chemistry domains (Figure 1a). The pipeline begins by retrieving descriptive text for each chemical from Wikipedia or, when unavailable, from PubMed abstracts. If exact name matches are not found, the pipeline expands queries using the top five synonyms from PubChem and repeats the retrieval process.^26,27^ Chemicals without retrievable text are assigned a fallback INFO label to prevent unsupported outputs. Full method details are provided in Text S1.

**Figure 1:**
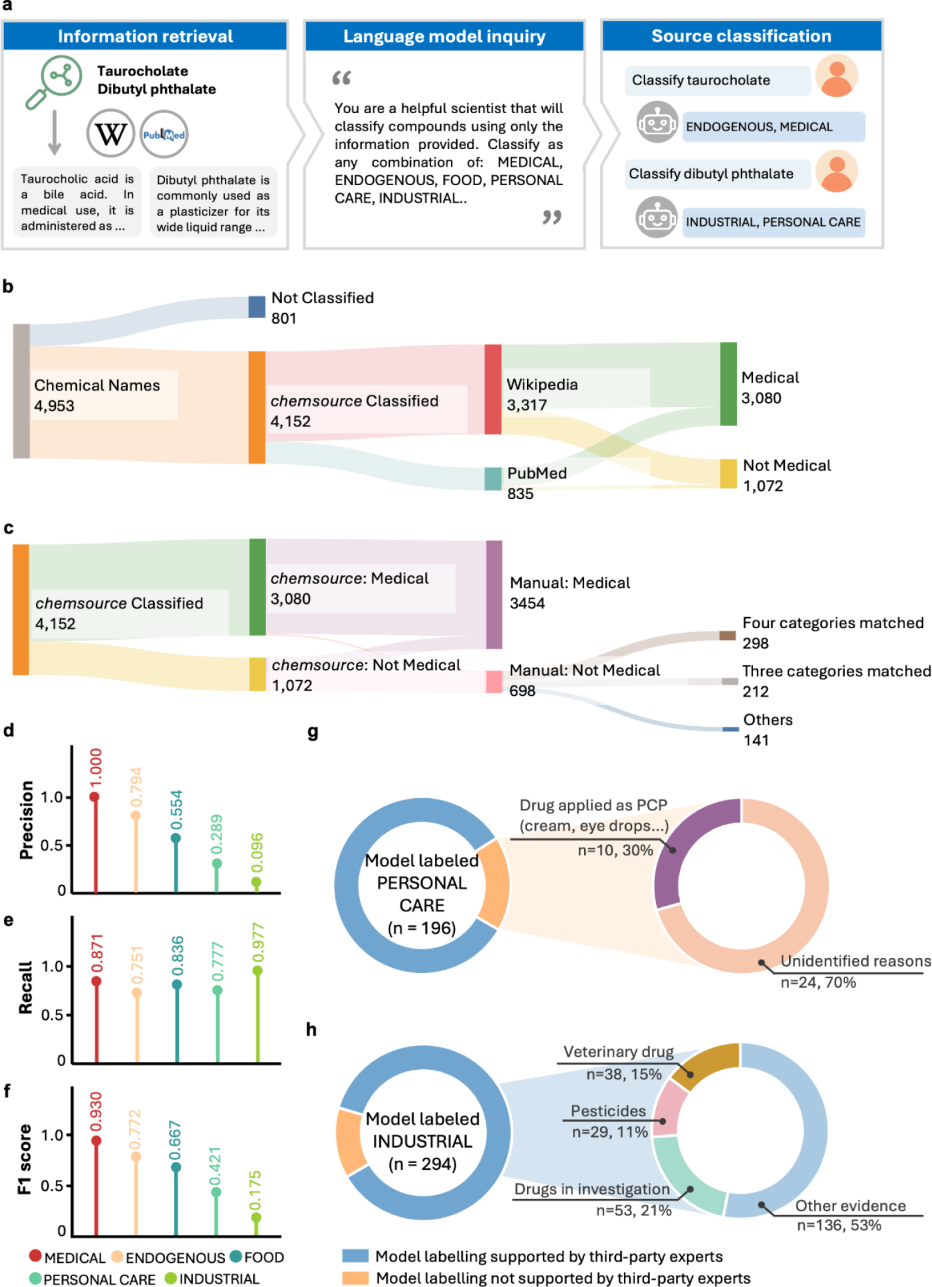
Overview of *chemsource* workflow and benchmark against manual labeling. a, Diagrams of the *chemsource* method pipeline. Textual information on the chemicals is retrieved preferentially from Wikipedia page body text, with the top 3 abstracts from PubMed as an alternative source if no information is found on Wikipedia. After minimal processing, the text retrieved is pushed to the LLM along with a predefined prompt for chemical source classification. b, Distribution of *chemsource* classifications for 4,953 compounds with manual labels of exposure sources. The widths of the bars and lines reflect the number of chemicals in each case. c, Comparison of *chemsource* classifications with the manual labels. d-f, Precision (d), recall (e), and F1 score (f) of *chemsource* classification in each exposure category, compared with manual labels. g, Third-party review of PERSONAL CARE labels by *chemsource* that were not assigned manually (*n* = 196). For 83% of cases, independent experts found supporting evidence of use in personal care products. For the remaining 17% of cases, 30% of the compounds were administered in forms that may have caused misclassification by *chemsource* (e.g., drugs used topically as creams or eye drops). h, Retrospective review of INDUSTRIAL-labeled compounds (*n* = 294 model labels not assigned manually). In 87% of cases, experts validated industrial use. Misalignment often involved edge cases such as veterinary drugs, pesticides, and investigational or withdrawn drugs, which *chemsource* correctly classified based on the prompt definition.

### Prompt Engineering

Chemical classification in *chemsource* is performed by querying an LLM with a structured prompt appended by the retrieved text. The default prompt incorporates established prompt-engineering practices, including role-based prompting, clear task framing, explicit category definitions, and repeated reminders to avoid unsupported assumptions.^19,28^ The default prompt contains three main components (see Text S2 for a full copy of the prompt):

#### Task Introduction

The model is role-prompted as a “scientist” and instructed to examine the entire retrieved text for evidence of five exposure-relevant categories (MEDICAL, ENDOGENOUS, FOOD, PERSONAL CARE, and INDUSTRIAL). As a result, a compound may receive multiple labels when evidence supports more than one exposure source, a single label when only one category is mentioned, or the fallback label INFO when no classification-relevant information is available. This approach captures compounds with mixed sources, such as those both endogenously produced and used as medications (e.g., progesterone, ursodiol).

#### Category Definition

Category definitions in the default prompt were optimized through expert-guided, iterative refinement using ∼10 representative examples for each exposure category (∼50 chemicals in total) including known edge cases with overlapping sources. This process focused on clarifying category boundaries, resolving ambiguous cases, and reducing overgeneralization with model outputs evaluated by domain knowledge and targeted literature review. Through iterative prompt testing, we found that precisely defined and mutually exclusive categories improve precision (see details in the Benchmarking against Manual Labeling section). Accordingly, in the default prompt, we defined the “MEDICAL” label as approved medications or compounds in late-stage clinical trials. “ENDOGENOUS” includes compounds produced by the human body, excluding essential nutrients that cannot be synthesized internally. “FOOD” refers to naturally occurring food compounds and food additives. “PERSONAL CARE” includes nonmedicated compounds used in skincare, beauty, or fitness products. “INDUSTRIAL” refers to synthetic compounds not used in medical, food, or personal care contexts (e.g., plasticizers, pesticides, and polymer ingredients). To reduce the likelihood of hallucinations or unsupported assumptions, the prompt also includes an “INFO” option, assigned when the retrieved text lacks sufficient information for confident classification.

#### Output Requirements

Output is requested as a plain-text, comma-separated list, which we found to be more stable than structured formats such as JSON. Before outputting, the prompt repeated the instruction to the LLM to base its classifications solely on the retrieved text to minimize reliance on prior model knowledge.

*chemsource* supports a range of language models with varying cost and performance profiles. For this study, GPT-4o was extensively evaluated and selected as the default model, because it showed strong accuracy and favorable cost at the time of evaluation. Temperature was by default set to 0 to promote deterministic output from the LLM.

### Performance Evaluation

We benchmarked *chemsource* against the GNPS Drug Library, which contains 4,953 compounds manually annotated using the same exposure categories.^25^ Overall accuracy and category-specific precision, recall, and F1 scores were evaluated. For the PERSONAL CARE and INDUSTRIAL categories, we performed an additional benchmark against the U.S. Environmental Protection Agency Chemical and Products Database (EPA CPDat). Method details are provided in Text S3.

### Application in Metabolomics Data Analysis

To demonstrate utility in metabolomics data analysis workflow, we applied *chemsource* to reanalyze eight public untargeted metabolomics data sets spanning human and animal tissues, environmental samples, and consumer product extracts.^30–34^ Raw data were processed using MZmine^35^ and annotated with the GNPS library^36^ prior to source labeling with *chemsource*. Method details are provided in Text S4 and Table S1.

## Results and Discussion

### Benchmarking against Manual Labeling

To evaluate the accuracy of the *chemsource* workflow, we benchmarked its output against the GNPS Drug Library,^25^ which includes 4,953 compounds manually annotated with exposure source labels. Our team curated these labels to distinguish chemicals used solely as medications from those also produced endogenously or found in food and consumer products (e.g., deoxycholic acid, an endogenous metabolite also used to treat bile acid synthesis disorders; lactitol, a food sweetener also used as a laxative). This manual curation took place over the course of 3 years and included a rigorous 3 months of extensive literature search pushed by three domain experts. In contrast, *chemsource* completed the task in approximately 3 h (using GPT-4o with RAG; no parallel query) with minimal expert intervention.

*chemsource* generated classification results for 4,152 compounds. Among these, 80% of the information texts were retrieved from Wikipedia and 20% from PubMed (Figure 1b). Classifications from *chemsource* matched the manual labels for 75% of the compounds across all five exposure categories. Most mismatches were minor, with 14% of the compounds differing by one exposure category. “MEDICAL-only” was the dominant exposure label in both the manual annotations (*n* = 3,454) and *chemsource* predictions (*n* = 3,080; Figure 1c), reflecting the drug-focused nature of compounds in the GNPS Drug Library and indicating that *chemsource* effectively captured this underlying distribution.

Note that during the manual curation, we assigned the “MEDICAL” exposure label to all compounds in the GNPS Drug Library based on documented medical uses in established drug knowledge bases, including DrugBank,^37^ DrugCentral,^38^ the Broad Institute Drug Repurposing Hub,^39^ and ChEMBL.^40^ To evaluate the performance of *chemsource* on nonmedical exposure sources, we excluded the “MEDICAL” label from both the manual and *chemsource* outputs, leaving 651 compounds with one or more nonmedical annotations. Under these conditions, *chemsource* matched the manual labels for 46% of the compounds, with an additional 33% differing by one exposure category (Figure 1c). To investigate the sources of mismatch, we further calculated the precision, recall, and F1 score for each exposure category. Recall was high across categories, ranging from 75% for ENDOGENOUS to 95% for INDUSTRIAL (Figure 1e). In contrast, precision was notably lower, particularly for INDUSTRIAL (10%) and PERSONAL CARE (30%) labels, compared to FOOD (55%), ENDOGENOUS (79%), and MEDICAL (100%, due to all compounds being manually labeled as MEDICAL; Figure 1d). Correspondingly, the F1 scores were 18% for INDUSTRIAL, 42% for PERSONAL CARE, and >67% for the rest of the categories (Figure 1f). These findings suggest that the primary source of disagreement was overlabeling by *chemsource* (i.e., false positives from *chemsource* or false negatives from manual curation), rather than failure to identify relevant exposure sources. We note that the benchmark data set contains much more MEDICAL labels (assigned to 3,622 compounds by *chemsource*) compared to ENDOGENOUS, FOOD, PERSONAL CARE, and INDUSTRIAL labels (assigned to 341, 576, 286, and 450 compounds, respectively); consequently, the performance evaluation is more comprehensive for MEDICAL than other exposure categories.

Two factors may explain the observed overclassification by *chemsource*: the model may have been overconfident, or the manual curation may have overlooked valid exposure sources. To investigate this, we had independent experts retrospectively and manually reviewed the PERSONAL CARE (*n* = 196) and INDUSTRIAL labels (*n* = 294; excluding compounds with overlapping PERSONAL CARE labels) assigned by *chemsource* but not present in the manual annotations (see expert notes for each compound in Tables S3, S4). For PERSONAL CARE, in 83% of cases (162 of 196), independent experts identified supporting evidence for personal care use through targeted web searches. Among the remaining 34 compounds, 10 were medications administered via topical formulations, such as creams, ear drops, or throat lozenges—modalities that may have led the model to mistakenly infer personal care use (Figure 1g; Table S3).

For INDUSTRIAL assignments, expert review confirmed industrial use for 87% of the compounds (256 out of 294 compounds; Figure 1h; Table S4). Interestingly, the expert identified 38 veterinary drugs that had been labeled as “MEDICAL-only” during manual curation but were reclassified by *chemsource* as “INDUSTRIAL”. This discrepancy reflects a design detail in our prompt: we explicitly defined “MEDICAL” as medications intended for human use, while “INDUSTRIAL” caught overspills of any synthetic compounds not used in medicine, food, or personal care products. This observation highlights that *chemsource* accurately followed prompt instructions and applied consistent logic in edge cases but also underscores that our original prompt definition lacked sufficient specificity for veterinary medications. Similarly, 29 pesticides were labeled as “INDUSTRIAL” by *chemsource* but had been annotated as “MEDICAL” in manual curation. The LLM also identified edge cases where the chemicals were used both as human and veterinary drugs (e.g., flubendazole, an anthelmintic;^41^ carprofen, a nonsteroidal anti-inflammatory drug used in human and veterinary medicine^42,43^ ), or as human drugs and pesticides (e.g., streptomycin, an antibiotic for humans and a pesticide for agriculture;^44,45^ permethrin, an antiparasite and an insecticide^46^), and it correctly labeled the exposure source as “MEDICAL, INDUSTRIAL” for these cases.

The expert review also identified 53 compounds with ambiguous or transitional medical status where *chemsource* assigned the label “MEDICAL, INDUSTRIAL”, such as withdrawn drugs (e.g., kanamycin,^47^ merbromin^48^), illicit substances and doping agents (e.g., acetomorphine,^49^ cardarine^50^), and early-stage therapeutic candidates (e.g., ebselen,^51^ calcimycin^52^). This labeling behavior likely stemmed from the strict definition of “MEDICAL” in the default prompt, which limited this category to drugs approved or in late-stage clinical trials. In rare cases (*n* = 4), misclassifications were due to incorrect text retrieval caused by polysemantic synonyms (Table S4). For example, the diuretic drug xipamide has the synonym “Aquaphor” in PubChem, a trade name used in Germany,^53^ leading *chemsource* to retrieve the Wikipedia page for the unrelated skincare brand also named “Aquaphor”.

Because the INDUSTRIAL labels involved multiple edge cases, we further assessed the reproducibility of *chemsource* by randomly selecting 20 INDUSTRIAL-labeled chemicals and repeating the classification 100 times. Seventeen out of the 20 compounds received INDUSTRIAL labels in all 100 runs, and the remaining three were labeled with INDUSTRIAL 88, 90, and 96 times (Table S2), indicating that *chemsource* produces reproducible outputs despite the probabilistic nature of LLMs.

To systematically verify our retrospective expert inspection and to more broadly evaluate how prompt design influences model performance, we developed three alternative prompts and reran *chemsource* on the benchmark data set (Figure S1). Prompt A relaxed the criteria for the MEDICAL category by removing the “approved or late-stage clinical trial” and “human use” requirements while narrowing the definition of INDUSTRIAL by explicitly excluding veterinary medications and pesticides. This modification increased INDUSTRIAL precision 1-fold from 10% to 22% (Figure S1), very consistent with our expert review that roughly half of the previous INDUSTRIAL “false positives” were veterinary drugs, pesticides, or early-stage pharmaceuticals (Figure 1h). Because the category definitions are mutually exclusive, broadening MEDICAL reduces spillover into INDUSTRIAL and improves INDUSTRIAL precision (Figure S1). As expected, recall remained nearly unchanged, because other industrial compounds with manual labels in the benchmark dataset were unaffected by the redefinition.

Prompt B retained the default category definitions for MEDICAL but removed the exclusivity constraint on INDUSTRIAL (i.e., allowing compounds used in medical, personal care, or food contexts to also receive INDUSTRIAL labels). This leads to a decrease in INDUSTRIAL precision from 10% to 7.5%, reflecting the expanded category boundary (Figure S1). Prompt C removed all category definitions entirely, requiring the model to infer meaning solely from the retrieved text. This resulted in overassignment: for example, ENDOGENOUS precision decreased from 79% to 65%, while recall increased from 75% to 82% (Figure S1).

Based on the above results, we concluded that the discrepancies for INDUSTRIAL and PERSONAL CARE categories were driven in large part by overlooked exposure sources during the manual annotation. Therefore, we conducted an additional evaluation of *chemsource* using EPA CPDat, a domain-specific source database that compiles chemical use information from publicly disclosed material safety data sheets and ingredient lists. Among the 336 benchmark compounds present in CPDat, *chemsource* showed good agreement after ontology harmonization (precision: 82–88%, recall: 47–76%, F1 scores: 61–79% for PERSONAL CARE and INDUSTRIAL categories; Figure S2, Table S5), providing independent support for the accuracy of *chemsource* in consumer product-related exposure contexts.

Together, the results demonstrate that prompt engineering directly governs the precision–recall balance: highly defined prompts increase precision by constraining model behavior, whereas looser prompts broaden recall at the cost of specificity. For chemical classification tasks, precision is generally more critical than maximal recall, and we therefore recommend including clear, mutually exclusive category definitions such as those in our default prompt. We also strongly recommend iterative prompt refinement through periodic manual spot-checking to continuously refine the category boundaries. Overall, our retrospective analyses demonstrate that *chemsource* is capable of performing standardized, evidence-based functional classification with accuracy comparable to, and in some cases exceeding, manual interpretation, particularly for edge-case compounds. Despite the need for occasional manual review, *chemsource* markedly reduces the time and labor required for functional chemical classification. Manual annotation requires ∼1–30 min per compound depending on complexity, which becomes prohibitive for large inventories (e.g., the U.S. Environmental Protection Agency’s CompTox database contains over one million exposure-relevant chemicals).^54^ In contrast, *chemsource* processes each compound in milliseconds, enabling scalable, automated classification with minimal human intervention.

### Comparison of RAG Schemes

*chemsource* was initially developed in April 2024 using OpenAI’s GPT-4, which at the time represented the state-of-the-art language model. Early testing demonstrated that incorporating RAG with curated text from Wikipedia and PubMed substantially improved classification accuracy compared to using LLMs alone. Since then, the LLM landscape has evolved rapidly, with the release of more capable models and cost-effective alternatives from multiple providers. To identify optimal configurations that balance performance and cost-efficiency, we rebenchmarked *chemsource* in April 2025 using a set of updated LLMs and RAG pipelines. Specifically, we evaluated four configurations: (a) GPT-4o-RAG, based on GPT-4o using our custom RAG pipeline with Wikipedia and PubMed retrieval; (b) DeepSeek-RAG, using DeepSeek-V3 with the same custom RAG pipeline;^24^ (c) GPT-Search, based on GPT-4.1-search-preview, which performs open-ended web retrieval using OpenAI’s internal search engine rather than relying on preretrieved Wikipedia or PubMed content (we used medium context length to balance retrieval depth and cost); and (d) GPT-no-RAG, using GPT-4.1 without any retrieval step, where classification is performed based solely on the model’s prior knowledge base (Figure 2a).

**Figure 2:**
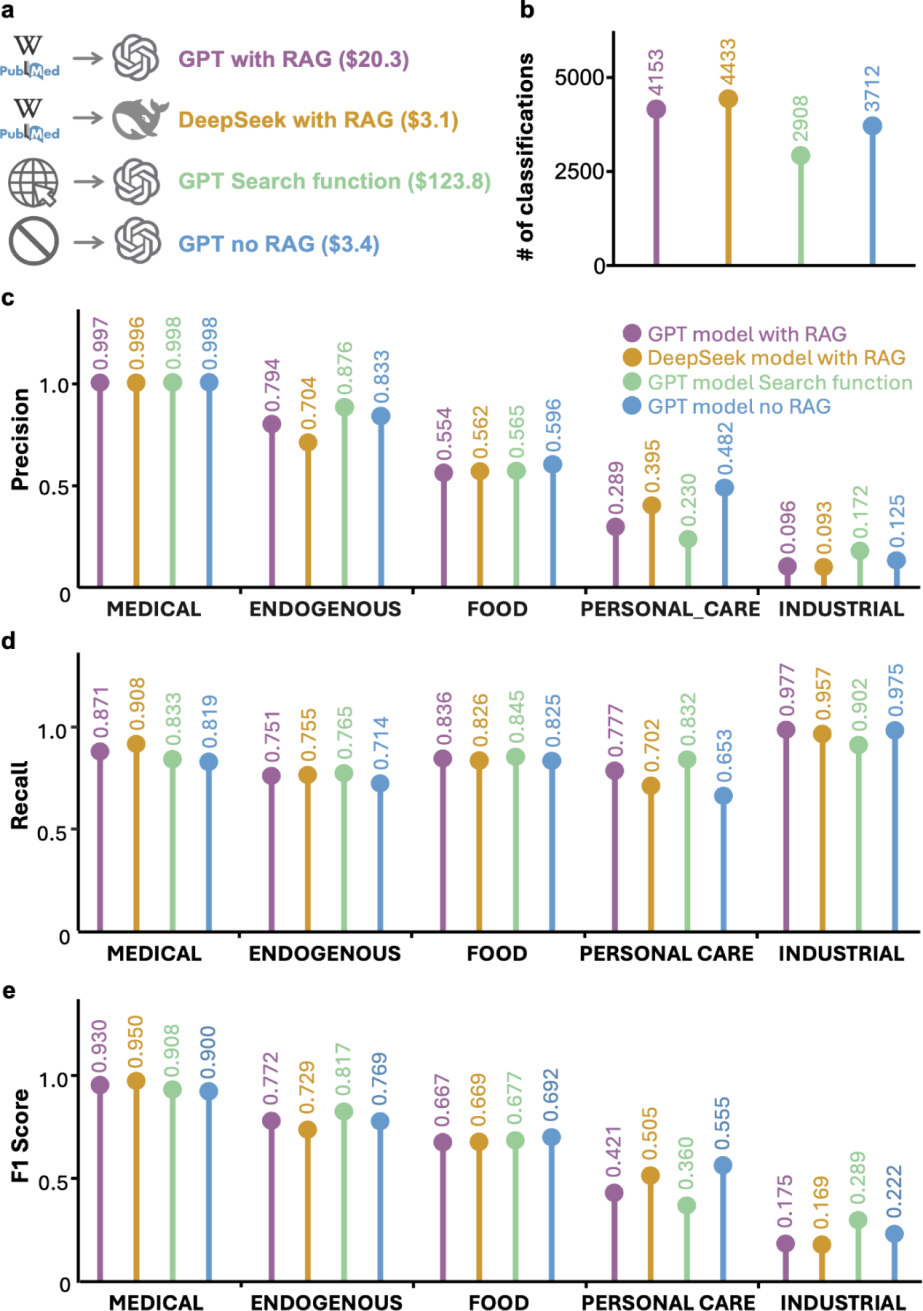
Comparison of retrieval-augmented generation (RAG) strategies and large language models (LLMs) for chemical classification tasks. a, Overview of the four configurations evaluated in April 2025. b, Total number of compounds classified under each configuration. c-e, Precision (c), recall (d), and F1 score (e) for each exposure category.

We found that GPT-4o-RAG, GPT-no-RAG, and DeepSeek-RAG performed at similar speeds, while the GPT-Search was ∼4 times slower (absolute time depends on network stability and parallelization; Table S6). Cost-wise, GPT-Search is ∼6 times more expensive than GPT-4o-RAG, and GPT-4o-RAG is ∼7 times more expensive than GPT-no-RAG and DeepSeek-RAG (Figure 2a). Our default RAG configuration enabled classification of more compounds compared to GPT-Search and GPT-no-RAG (number of classified compounds: GPT-4o-RAG, 4,153; GPT-Search, 2,908; GPT-no-RAG, 3,712; Figure 2b). This is likely due to the medium context length that we used in GPT-Search, restricting the availability of background information. Precision, recall, and F1 scores across exposure categories were generally consistent among all configurations (Figure 2c-e). We therefore recommend using RAG to ensure that model outputs are grounded in relevant, trackable information. However, for scenarios with limited budgets, direct prompting without retrieval (GPT-no-RAG) remains a viable, cost-effective alternative, offering approximately 7-fold cost savings (Figure 2a). In contrast, GPT-Search underperformed in both coverage and cost-efficiency, and we do not recommend its current implementation. However, future improvements in retrieval depth and cost may enhance its utility.

Finally, we observed that GPT-4o and DeepSeek-V3 yielded similar classification coverages and accuracy when given the same retrieved input text (Figure 2c-e). This suggests that modern LLMs are generally effective at interpreting high-quality retrieved content and that further performance improvements may depend more on enhancing the quality and relevance of retrieved content than on adopting newer or more powerful model architectures. The cost of running *chemsource* can be reduced ∼7-fold by using DeepSeek-V3, a trend that we expect to continue as open-source LLM ecosystems evolve (Figure 2a).

### Application in Metabolomics Data Analysis

To demonstrate the utility of *chemsource* in metabolomics data analysis, we applied the workflow to eight public untargeted metabolomics data sets encompassing a diverse set of sample types. These included three human biospecimens (feces, plasma, and brain samples from Alzheimer’s disease cohorts),^30,31^ one mouse tissue data set,^32^ two environmental samples (dust from mattresses and surface swabs from the International Space Station),^33^ one data set of food extracts, and one for extracts of personal care products. We employed the GPT-4o-RAG configuration of *chemsource* and classified the exposure sources of chemicals annotated using the default GNPS Library, which includes not only the curated drug library but ∼1.3 million reference spectra of a broad range of molecules (see Upset plot in Figure S3 for classification results in each sample type).^34^

To visualize the exposure profiles across sample types, we calculated the relative abundance of each source category by summing the detection frequencies of chemicals classified into that category, followed by sum normalization (Figure 3a). Surface swabs from the International Space Station exhibited a higher relative abundance of personal care chemicals, likely reflecting the routine use of disinfectants and hygiene products in spacecraft environments.^33^ Other sample types, particularly human biospecimens, showed broadly similar profiles, with higher contributions from endogenous and food-derived chemicals (Figure 3a).

**Figure 3:**
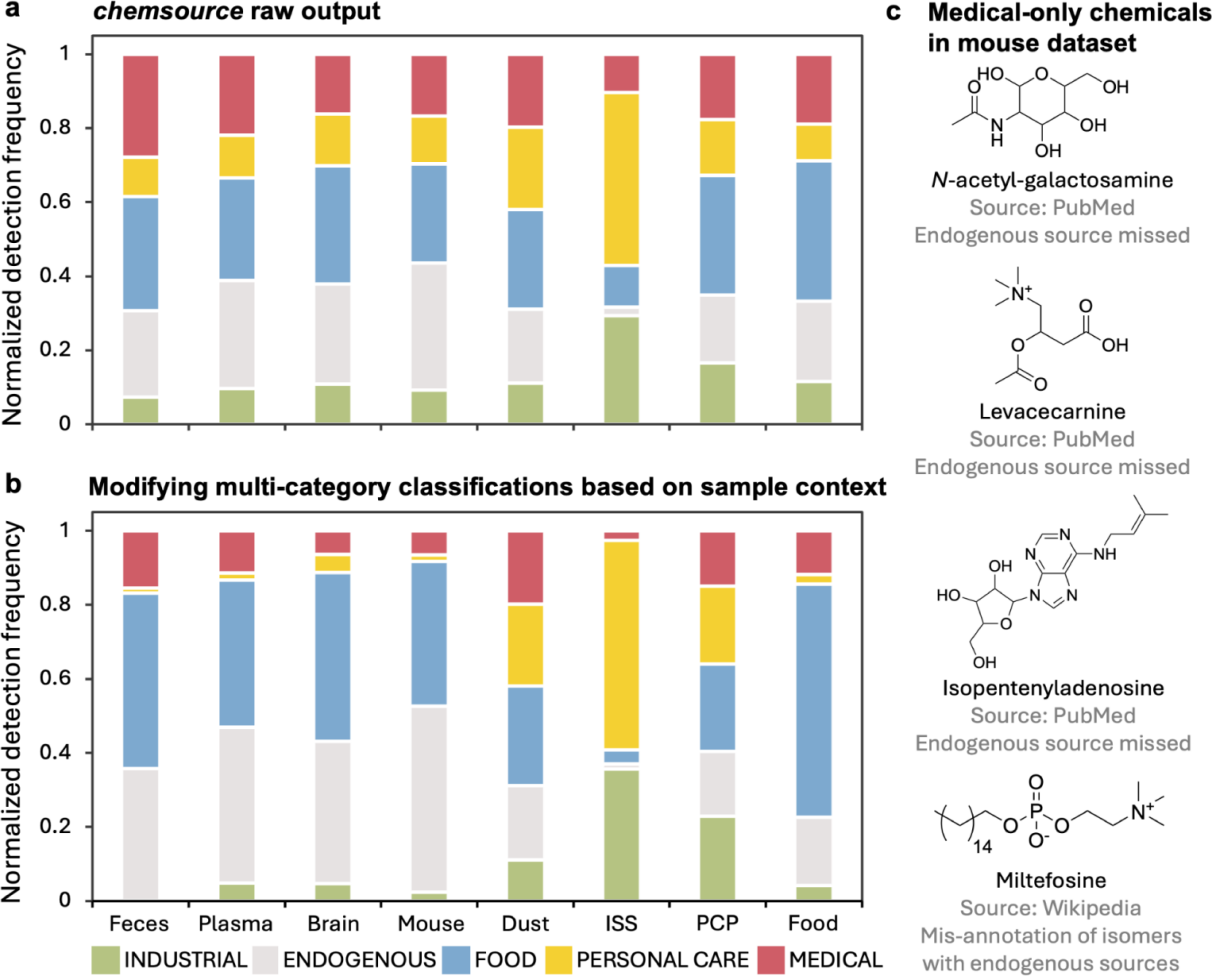
Application of *chemsource* to annotate exposure profiles in different sample types using public untargeted metabolomics data sets. a, Exposure profiles across eight public metabolomics data sets determined by *chemsource* using the GPT-4o-RAG configuration. Data sets span a range of sample types: human feces, plasma, brain tissue (from Alzheimer’s disease cohorts), mouse tissues, mattress dust, surface swabs from the International Space Station (ISS), personal care product extracts (PCP), and food extracts (Food). Exposure source distributions were computed by summing the detection frequencies of annotated compounds in each category followed by normalization. b, Refined exposure profiles after applying context-specific modifications to resolve overrepresentation from multicategory annotations. In biospecimen data sets (i.e., plasma, feces, brain, and mouse tissues), multicategory annotations were restricted to FOOD and ENDOGENOUS; in personal care and ISS data sets, annotations were restricted to PERSONAL CARE and INDUSTRIAL. c, Compounds with high detection frequencies labeled as MEDICAL-only in the mouse data set, annotated alongside their retrieval sources and the likely reasons other sources were missed.

Compounds assigned to multiple source categories may be overrepresented in the contexts of specific data sets. For instance, phenylalanine was classified as “FOOD, MEDICAL, and PERSONAL CARE”. While this reflects its known multidomain usage, within the context of the food extract data set (solvent extraction of food ingredients), phenylalanine is most appropriately considered as FOOD-only. To address this issue, we implemented a context-based refinement procedure to adjust classification outputs based on sample types. Specifically, for the human biospecimens and mouse data sets, multicategory assignments with ENDOGENOUS and/or FOOD labels were reduced to ENDOGENOUS and/or FOOD-only (i.e., removing medical, personal care, and industrial labels when the compounds can be sourced from host metabolism or food). In the food data set, compounds assigned with multiple categories including FOOD were reduced to FOOD-only. For the personal care product and International Space Station data sets, we retained only PERSONAL CARE and/or INDUSTRIAL labels for multicategory assignments, as these compounds are likely direct additives. We did not apply context-based modifications to the dust data set, which does not have a dominant expected exposure source.

After context-based refinement, distinct and more interpretable exposure profiles emerged across data sets (Figure 3b). As expected, the ISS samples remained enriched in personal care and industrial chemicals, and the food data set was dominated by food-related compounds. The mouse data set exhibited minimal presence of medical compounds, but the persistence of several MEDICAL-only labels initially appeared counterintuitive for an untreated animal model. A closer examination revealed that these labels primarily originated from less-documented endogenous metabolites with potential pharmaceutical applications, such as *N*-acetylgalactosamine, acetylcarnitine, and isopentenyladenosine, all detected at high frequencies (>60%; Figure 3c). For these compounds, the retrieved text came from PubMed abstracts, which emphasize biomedical usage and often provide limited coverage of endogenous biochemical roles. We also identified instances in which MS/MS library annotations were likely misassignments of structural isomers. For example, miltefosine, a widely used anthelmintic drug, was detected with 45% frequency. Although miltefosine itself has no known endogenous source, its ether lipid scaffold is shared by endogenous phosphocholine analogues (Figure 3c), making it likely that an endogenous isomer was incorrectly matched to the miltefosine reference spectra. Together, these observations show that biases in retrieval sources or spectral annotations can propagate through the *chemsource* and influence exposure source profiles. We therefore recommend careful review of PubMed-only classifications, particularly in contexts where pharmaceutical exposure is not expected.

## Limitations

One limitation of this study arises from the benchmarking data set, as the manual annotations in the GNPS Drug Library contain errors and omissions. As noted earlier, the high recall but low precision for the PERSONAL CARE and INDUSTRIAL categories largely reflects missed labels in the manual annotation. Despite two rounds of manual review during the development of the GNPS Drug Library, some misclassifications are unavoidable at this scale, and expert judgments can vary depending on the information sources consulted. Accordingly, agreement rates between *chemsource* and manual labels should be interpreted with the understanding that the “ground truth” is imperfect, as is typical for large-scale manual curation.

More broadly, this limitation reflects a structural challenge in chemical source annotation: while several high-quality databases exist, no single resource comprehensively captures exposure-source classifications across endogenous, dietary, pharmaceutical, personal care, and industrial contexts. Existing databases are typically domain-specific and often overlap in scope; for example, HMDB (Human Metabolome Database)^55–59^ and ChEBI (Chemical Entities of Biological Interest)^60^ include drugs and food-derived compounds because they are present endogenously in humans, whereas consumer product databases emphasize usage rather than biological relevance.^29,54^ Consequently, database-derived labels may be internally consistent within a given domain but are not universally applicable to metabolomics interpretation workflows. Rather than enforcing a fixed ontology, *chemsource* is designed to flexibly integrate diverse knowledge sources and user-defined classification schemes, allowing exposure-source labeling to be adapted to specific data sets and research questions.

The performance of *chemsource* is inherently dependent on the quality and availability of the input text. Because Wikipedia and PubMed contain more information about well-studied and biomedical chemicals, classification success rates are naturally higher for these compounds. In contrast, discontinued drugs, early-stage experimental chemicals, obscure natural products, and other poorly documented compounds are more likely to lack sufficient detail for confident labeling. In practice, positive classifications by *chemsource* are generally accurate and grounded in trackable evidence, whereas the absence of particular exposure sources should be interpreted with caution.

A further limitation stems from the prompt design and user-defined category definitions. Ambiguous or overly broad prompts can lead to misaligned or excessive multicategory assignments, whereas overly narrow definitions may exclude relevant compounds. We recommend defining clear, mutually exclusive categories and iteratively refining prompts through targeted evaluations. *chemsource* can also be extended by applying additional prompts to subclassify compounds for specific research needs; we provided example prompts in the Supporting Information to subdivide MEDICAL compounds by therapeutic area, distinguish FOOD constituents from additives, and annotate INDUSTRIAL chemicals by specific functions (Text S5).

Finally, *chemsource* inherits limitations from the LLMs on which it relies. These include run-to-run variability, sensitivity to prompt phrasing, occasional formatting inconsistencies, and minor typographical errors (e.g., “ENDOGENEOUS” misspelled as “ENDOGENOUS” one time in the GNPS Drug Library benchmark). Use of high-performing models may also be constrained by API costs, rate limits, or service availability. To mitigate these issues, the *chemsource* documentation includes examples for using alternative providers such as DeepSeek (a low-cost option) and Google Gemini (which offers free, rate-limited access). We recommend performing large-scale classification asynchronously to reduce delays associated with API rate limits. For advanced users, *chemsource* can be paired with offline, open-source LLMs (e.g., Llama, Mistral, Qwen, DeepSeek) via frameworks such as Ollama, fully eliminating the dependence on external APIs and improving long-term accessibility and reproducibility.

## Conclusion

*chemsource* provides a flexible and scalable framework for chemical classification by integrating retrieval-augmented text evidence with large language models. By avoiding fixed ontologies and querying static databases, *chemsource* enables customizable, evidence-traceable chemical labeling adaptable to diverse metabolomics and exposomics applications. Performance largely depends on the prompt design, highlighting the importance of clear, mutually exclusive category definitions and iterative prompt refinement. Continued advances in language models, prompt engineering practices, and domain-specific knowledge resources are expected to further improve the performance of LLM-based chemical classification.

## Supplementary Material





## Data Availability

*chemsource* is available as a Python package from PyPI, the Python Package Index, at https://pypi.org/project/chemsource/, or from GitHub at https://github.com/prajitrr/chemsource. Documentation and examples of *chemsource* usage are available at https://chemsource.readthedocs.io/en/latest/index.html. Code for data analysis of the manuscript is available at https://github.com/prajitrr/chemsource-paper. Untargeted metabolomics data sets to demonstrate *chemsource* application are publicly available in GNPS/MassIVE under the accession numbers MSV000095418 (human feces), MSV000096884 (human plasma), MSV000093059 (mouse tissue), MSV000079274 (mattress dust), MSV000094202 (surface swabs from the International Space Station), MSV000096584 (food extracts), and MSV000095003 (personal care product extracts).

## Notes

Methods for information retrieval in *chemsource* workflows (Text S1); Default system prompt used by *chemsource* (Text S2); Methods for performance evaluation against GNPS Drug Library and EPA CPDat (Text S3); Methods for compound annotation for public metabolomics data sets (Text S4); Example prompts for subclassifying MEDICAL, FOOD, and INDUSTRIAL labels (Text S5); Evaluation of alternative prompt designs for *chemsource* classification (Figure S1); Evaluation of *chemsource* classification against EPA CPDat for INDUSTRIAL and PERSONAL CARE labels (Figure S2); Upset plots for classification results of annotated compounds in public metabolomics data sets (Figure S3) (PDF)
Feature extraction parameters for public metabolomics data sets in MZmine (Table S1); Results of repetitive queries to test model reproducibility (Table S2); Third-party review of PERSONAL CARE labels by chemsource (Table S3); Third-party review of INDUSTRIAL labels by chemsource (Table S4); Benchmark chemsource against CPDat database (Table S5); Runtime estimates for classifying 5,000 compounds (Table S6); chemsource results and manual labeling for all chemicals in GNPS Drug Library (Table S7) (XLSX)
