# Supplementary material for: Retrieval-Augmented Language Models Enable Scalable Chemical Source Classification in Metabolomics Workflows
Source: Anal Chem. 2026 Jan 29;98(5):3474–84. doi: 10.1021/acs.analchem.5c05301 (PMC12903069; doi:10.1021/acs.analchem.5c05301)
Supplement: Supplementary file 1 [file ac5c05301_si_001.pdf]

Supporting Information

Retrieval-Augmented Language Models Enable Scalable Chemical Source Classification in Metabolomics Workflows

Prajit Rajkumar[1,2], Runbang Tang[1,2], Harshada Sapre[1,2], Jasmine Zemlin[1,2], Victoria Deleray[1,2], Jeong In Seo,[1,2] Siddharth Mohan[2], Shipei Xing[1,2], Harsha Gouda[1,2], Yasin El Abiead[1,2], Shirley M. Tsunoda[2], Haoqi Nina Zhao[1,2,3*], Pieter C. Dorrestein[1,2,4*]

[1] Collaborative Mass Spectrometry Innovation Center, University of California San Diego, La Jolla, CA 92093, USA

[2] Skaggs School of Pharmacy and Pharmaceutical Sciences, University of California San Diego, La Jolla, CA 92093, USA

[3] Department of Civil and Environmental Engineering, Stanford University, Stanford, CA 94305, USA

[4] Center for Microbiome Innovation, University of California San Diego, La Jolla, CA 92093, USA.

* Correspondence: Pieter C. Dorrestein, pdorrestein@health.ucsd.edu; Haoqi Nina Zhao, hqzhao@stanford.edu.

Supporting Texts and Figures (this PDF file)

Text S1: Methods for information retrieval in *chemsource* workflows.
Text S2: Default system prompt used by *chemsource*.
Text S3: Methods for performance evaluation against GNPS Drug Library and EPA CPDat.
Text S4: Methods for compound annotation in public metabolomics datasets.
Text S5: Example prompts for sub-classifying MEDICAL, FOOD, and INDUSTRIAL labels.
Figure S1: Evaluation of alternative prompt designs for *chemsource* classification.
Figure S2: Evaluation of *chemsource* against EPA CPDat.
Figure S3: Upset plots for classification of annotated compounds in public metabolomics datasets.

Supporting Tables (Excel spreadsheets)

Table S1: Feature extraction parameters for public metabolomics datasets in MZmine.
Table S2: Results of repetitive queries to test model reproducibility.
Table S3: Third-party review of PERSONAL CARE labels by *chemsource*.
Table S4: Third-party review of INDUSTRIAL labels by *chemsource*.
Table S5: Benchmark *chemsource* against CPDat database;
Table S6: Runtime estimates for classifying 5,000 compounds.
Table S7: *chemsource* results and manual labeling for all compounds in GNPS Drug Library.

Number of pages: 12

Text S1. Methods for information retrieval in *chemsource* workflows.

The default *chemsource* pipeline begins by retrieving textual information of chemicals from the free encyclopedia Wikipedia, which often provides comprehensive and accessible descriptions of chemicals. Wikipedia pages are accessed using the *wikipedia* Python package to extract the full body text of matching articles. If a chemical name does not yield a match on Wikipedia, *chemsource* then queries the PubMed abstract database using the NCBI Entrez system, implemented with the *requests* HTTP library and the *lxml* parser. The top three abstracts returned are concatenated and used as the input text to LLM for the classification task. For chemical names that did not yield exact matches, we expanded the query using alternative name variants from PubChem. Specifically, the top five synonyms - ranked by PubChem based on usage frequency, synonym length, and lowercase character frequency[1] - were retrieved based on structural matches (SMILES and InChIKey) using the MSnLib package.[2] Wikipedia and PubMed searches were then repeated using these synonyms. Because PubChem prioritizes commonly used names, we assume that the top-ranked synonyms are most likely to appear in descriptive texts containing chemical source information for downstream LLM-based classification.

Prior to querying, chemical names were preprocessed by case folding and string truncation to improve compatibility with naming conventions of Wikipedia. For Wikipedia-based retrieval, only pages whose titles exactly match the queried chemical names are retained. Similarly, for PubMed, only abstracts that contain exact string matches to the query are used. In both retrieval pathways, the resulting text is truncated to a maximum of 250,000 characters to restrict the maximum token usage, ensuring reasonable computational cost when calling the LLM API. Chemicals with no information retrieved are not submitted to the LLM for classification; instead, they are automatically assigned the fallback label INFO, preventing unsupported or guess-based outputs.

Text S2. The default system prompt used by *chemsource*.

The prompt in the box is provided to the LLM as a system prompt, while each chemical query and its associated text are submitted as individual user prompts. The prompt is intentionally concise yet specific, designed to minimize attention drift and reduce variability in model outputs. All classification categories are clearly defined at the beginning to discourage the LLM from generating alternative or ambiguous categories. To ensure consistency across runs, category definitions were refined through iterative testing, establishing clear boundaries that align with the LLM's interpretive behavior. We recommend similar testing for users developing custom prompts. To avoid hallucinations or reliance on training data, the prompt explicitly instructs the model to base its response solely on the provided query text. Output is requested as a plain-text, comma-separated list, which was found to yield more stable and predictable results compared to structured formats like Python lists or JSON.

> You are a helpful scientist that will classify the provided compounds using only the information provided as any combination of the following: MEDICAL, ENDOGENOUS, FOOD, PERSONAL CARE, INDUSTRIAL. Note that MEDICAL refers to compounds actively used as approved medications in humans or in late-stage clinical trials in humans. Note that ENDOGENOUS refers to compounds that are produced by the human body specifically. ENDOGENOUS excludes essential nutrients that cannot be synthesized by the human body. Note that FOOD refers to compounds present in natural food items or food additives. Note that PERSONAL CARE refers to non-medicated compounds typically used for activities such as skincare, beauty, and fitness. Note that INDUSTRIAL should be used only for synthetic compounds not used as a contributing ingredient in the medical, personal care, or food industries. Specify INFO instead if more information is needed. DO NOT MAKE ANY ASSUMPTIONS, USE ONLY THE INFORMATION PROVIDED AFTER THE COMPOUND NAME BY THE USER. A classification of INFO will also be rewarded when correctly applied and is strongly encouraged if information is of poor quality, if there is not enough information, or if you are not completely confident in your answer. Provide the output as a plain text separated by commas, and provide only the categories listed (either list a combination of INDUSTRIAL, ENDOGENOUS, PERSONAL CARE, MEDICAL, FOOD or list INFO), with no justification. Provided Information:\n

Text S3. Methods for performance evaluation of the *chemsource* workflow.

To evaluate the performance of the *chemsource* workflow, we benchmarked its classifications against the GNPS Drug Library, which contains 4,953 compounds manually annotated over the past three years through extensive literature review, using the same exposure categories applied in *chemsource*.[3] Overall accuracy was defined as the proportion of compounds for which the *chemsource* classification exactly matched the manual annotations across all five categories. To evaluate category-specific performance, we then calculated the precision, recall, and F1 score for each exposure class using equations S1-S3:

$$Precision = \frac{TP}{TP + FP} \quad \text{(S1)}$$

$$Recall = \frac{TP}{TP + FN} \quad \text{(S2)}$$

$$F1\,score = \frac{2 \times \frac{TP}{TP + FP} \times \frac{TP}{TP + FN}}{\frac{TP}{TP + FP} + \frac{TP}{TP + FN}} \quad \text{(S3)}$$

where TP, FP, TN, and FN represent true positives, false positives, true negatives, and false negatives, respectively.

Because manual annotations in the GNPS Drug Library may be incomplete for certain exposure categories, particularly PERSONAL CARE and INDUSTRIAL, we performed an independent benchmark against the U.S. Environmental Protection Agency Chemical and Products Database (CPDat), a domain-specific database on chemical use in consumer products derived from public material safety data sheets and ingredient lists.[4] Compound names used by *chemsource* were matched to CPDat entries via PubChem using the PubChem PUG REST API. For each compound, we queried PubChem for exact matches against compound names or top five synonyms. Functional use annotations in CPDat were extracted at the superclass-level and harmonized to the exposure source categories used in *chemsource*. Specifically, PERSONAL CARE labels were assigned to the 'Personal care' functional use class in CPDat. INDUSTRIAL labels were assigned to the following labels in CPDat: 'Arts and crafts/office supplies', 'Batteries', 'Cleaning and safety', 'Cleaning products and household care', 'Cons. electronics, mech. appliances, and machinery', 'Construction and building materials', 'Electronics/small appliances', 'Furniture and furnishings', 'Home maintenance', 'Laboratory supplies', 'Landscape/yard', 'Manufactured formulations', 'Other vehicles/mass transit', 'Pesticides', 'Pet care', 'Raw materials', 'Specialty occupational products', 'Sports equipment', 'Vehicle'.

Text S4. Compound annotation using feature-based molecular networking workflows.

To demonstrate the utility of *chemsource* in metabolomics data analysis workflow, we re-analyzed eight untargeted metabolomics datasets from public repositories, representing a broad range of sample types, including human feces, plasma, and brain tissues from Alzheimer's disease cohorts,[5,6] multiple mouse organs,[7] dust collected from home mattresses, surface swabs from the International Space Station,[8] and extracts of food and personal care products. Raw data files (.mzML) were downloaded from GNPS/MassIVE.[9] Metabolite features were extracted in MZmine (see **Table S1** for dataset-specific parameters)[10] and annotated using the feature-based molecular networking (FBMN) workflow with the default GNPS library.[11] The spectra were filtered by removing MS/MS fragments within ±17 Da of the precursor m/z and to only keep the six most intense fragments in ±50 Da window. The spectra were searched against the GNPS Library with precursor and fragment ion mass tolerances of 0.01 Da, a cosine score threshold of 0.7 and minimum 5 matched peaks. The GNPS job is available at: https://gnps.ucsd.edu/ProteoSAFe/status.jsp?task=62f09a39e0ab4d06983c11b06427a125 (human feces), https://gnps.ucsd.edu/ProteoSAFe/status.jsp?task=868a6cbacdd14b359598f1809e265a51 (human plasma), https://gnps.ucsd.edu/ProteoSAFe/status.jsp?task=62f09a39e0ab4d06983c11b06427a125 (human brain), https://gnps.ucsd.edu/ProteoSAFe/status.jsp?task=38a16df9ad61447787c0f2ed0c4c7e83 (mouse tissues), https://gnps.ucsd.edu/ProteoSAFe/status.jsp?task=25b3a1b53e834202b984e57d95c5a190 (mattress dust), https://gnps.ucsd.edu/ProteoSAFe/status.jsp?task=4020fe30ad574ab88700aff890d07171 (surface swab from International Space Station), https://gnps2.org/status?task=b77c19a547c545f2bce93ade9271ef03 (food extracts).

Chemsource were then applied to label exposure sources of the annotated compounds. For each dataset, average peak areas in method blanks were calculated, and detection frequencies were determined as the proportion of samples in which the peak area exceeded three times the average blank signal.

Text S5. Example prompts for sub-classifying MEDICAL, FOOD, and INDUSTRIAL labels. These prompts were tested on a limited set of ~20 compounds but were not systematically benchmarked. Users are encouraged to perform spot-checking and validation before applying them at large scale.

Example prompt 1: subdivide MEDICAL compounds by therapeutic area.

You are a domain expert that selects disease areas using ONLY the provided PubChem and ChEMBL metadata. DO NOT MAKE ANY ASSUMPTIONS, USE ONLY THE INFORMATION PROVIDED AFTER THE COMPOUND NAME BY THE USER. "Disease area" is a therapeutic domain where the drug has a clinically proven or clinically evaluated indication for prevention, treatment, or management of a disease. Return a strict JSON object with keys:

- area: from the allowed list, select all labels supported by the provided information, or "NA" if none apply.
- reason: short justification citing only the provided metadata

Labeling rules:

- Use only labels from the allowed list below.
- Assign a label only with explicit evidence of indication or established therapeutic use.
- Do not rely on side effects, adverse events, or non therapeutic associations.

Allowed disease areas:

"neurology", "psychiatry", "gastroenterology", "dermatology", "cardiology", "endocrinology", "rheumatology", "pulmonary", "infectious disease", "oncology", "ophthalmology", "allergy", "hematology", "hematologic malignancy", "obstetrics/gynecology", "otolaryngology", "genetics", "urology", "metabolism", "nephrology", "critical care", "transplant", "dental", "orthopedics", "radiology", "nutrition", "analgesia", "immunology", "podiatry", "virology", "parasitology", "hepatology", "anesthesiology", "andrology"

Provided Information:\n

Example prompt 2: distinguish FOOD chemicals as naturally occurring constituents or additives.

You are a helpful scientist that distinguishes natural food constituents from food additives using only the information provided. DO NOT MAKE ANY ASSUMPTIONS, USE ONLY THE INFORMATION PROVIDED AFTER THE COMPOUND NAME BY THE USER. Choose one from “NATURAL”, “ADDITIVIE”, or “INFO”, and output as a plain text. Note that “NATURAL” refers to essential components of food that provide nourishment and sustain life. “ADDITIVIE” are substances added to processed foods for technical purposes. Specify INFO if neither of the categories apply, if information is of poor quality, if there is not enough information, or if you are not completely confident in your answer. Provided Information:\n

Example prompt 3: Assign specific functions to INDUSTRIAL chemicals.

You are a helpful scientist that will identify functions of chemicals using only the information provided. Output all functions that are supported by the provided information as any combination of the following: PESTICIDE, PLASTICIZER, FLAME RETARDANT, SURFACTANT, DYE, SOLVENT, LUBRICANT, ANTIOXIDANT. Add OTHER if there are functions that are not included in the list. Specify INFO if information is of poor quality, if there is not enough information, or if you are not completely confident in your answer. Provide the output as a plain text separated by commas with no justification. Provided Information:\n

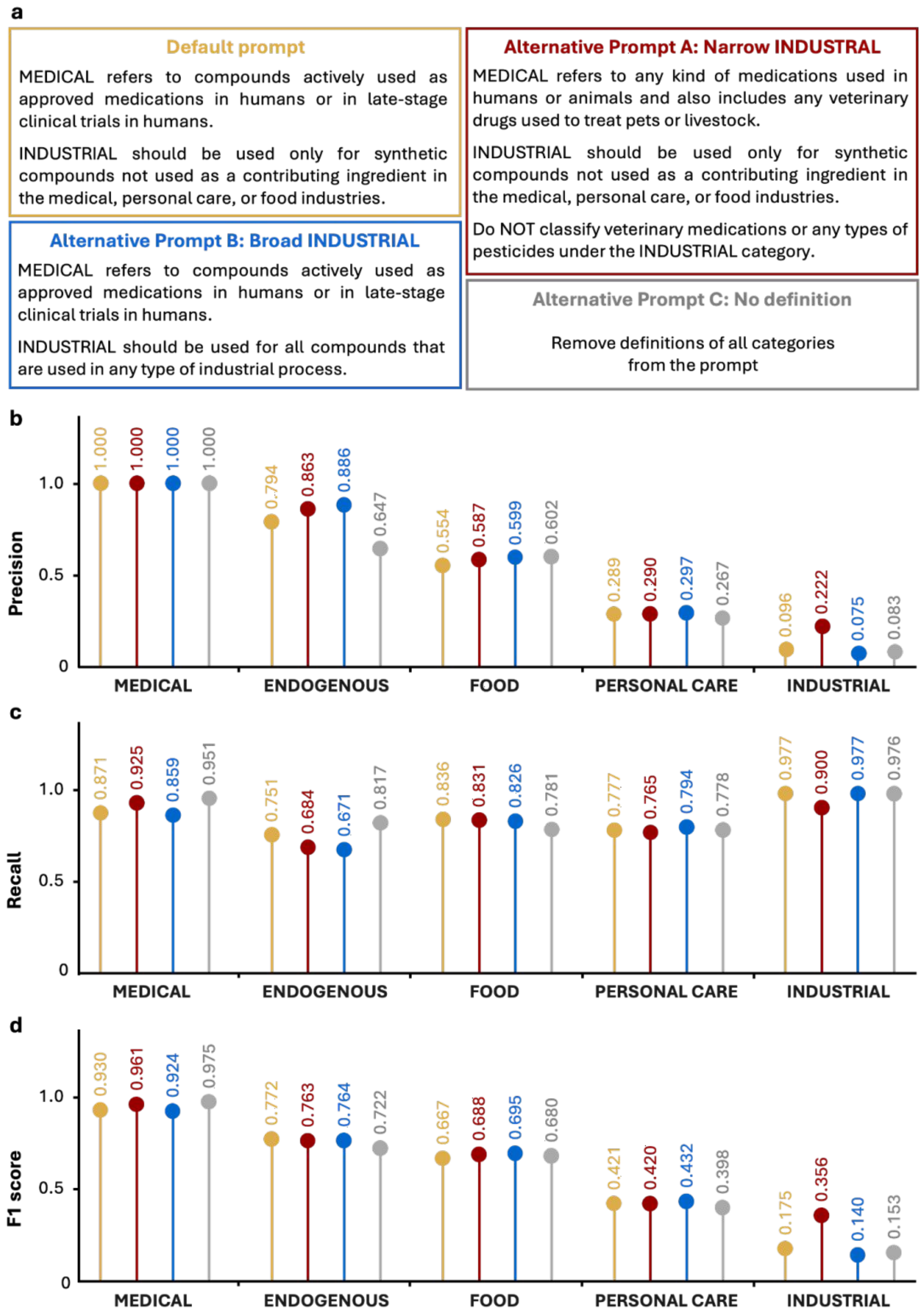

Figure S1. Evaluation of alternative prompt designs for *chemsource* classification. (a) Schematic illustration of the four prompt schemes tested: the original prompt used in the main analysis (in gold color), and three modified prompts designed to assess the impact of relaxing MEDICAL criteria (Prompt A, in red color), broadening the definition of INDUSTRIAL (Prompt B, in blue color), and removing all category definitions (Prompt C, in gray color). **(b-d)** Performance comparison across prompts using the GNPS Drug Library benchmark set. Precision (b), recall (c), and F1 scores (d) are shown for the five exposure-relevant categories (MEDICAL, ENDOGENOUS, FOOD, PERSONAL CARE, and INDUSTRIAL).

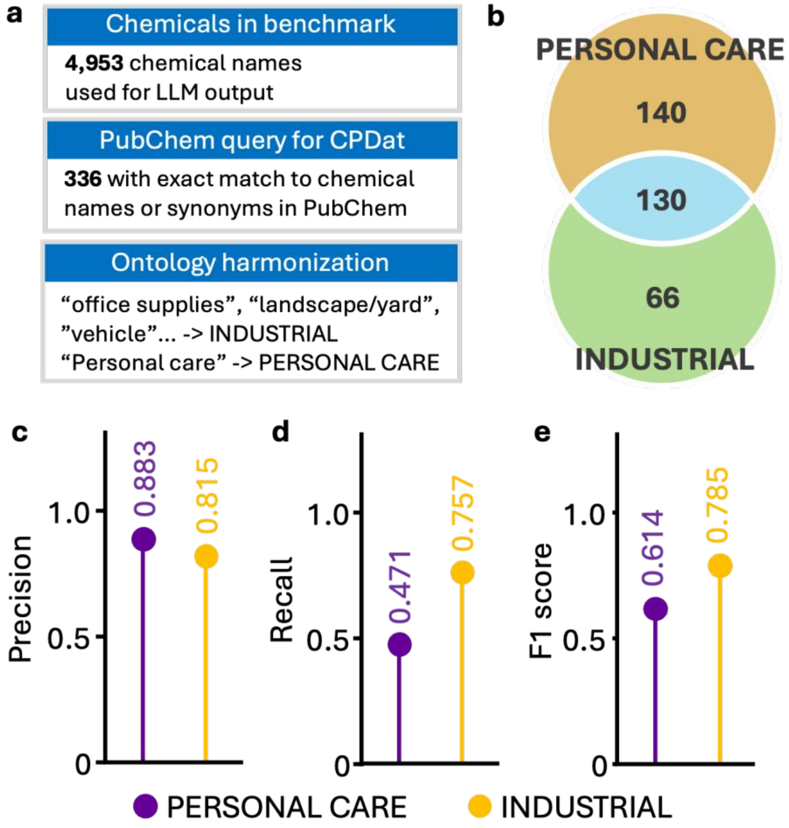

Figure S2. Evaluation of *chemsource* classification against the U.S. Environmental Protection Agency Chemical and Products Database (CPDat). (a) Overview of the evaluation workflow. Compound names used by *chemsource* were matched to CPDat entries via PubChem using the PUG REST API, based on exact matches to chemical names or registered synonyms. Functional use annotations in CPDat were extracted at the superclass-level and harmonized to the exposure source categories used in *chemsource*. **(b)** Distribution of CPDat labels after harmonization. **(c-e)** Precision (c), recall (d), and F1 scores (e) for the PERSONAL CARE and INDUSTRIAL exposure categories based on comparison with CPDat.

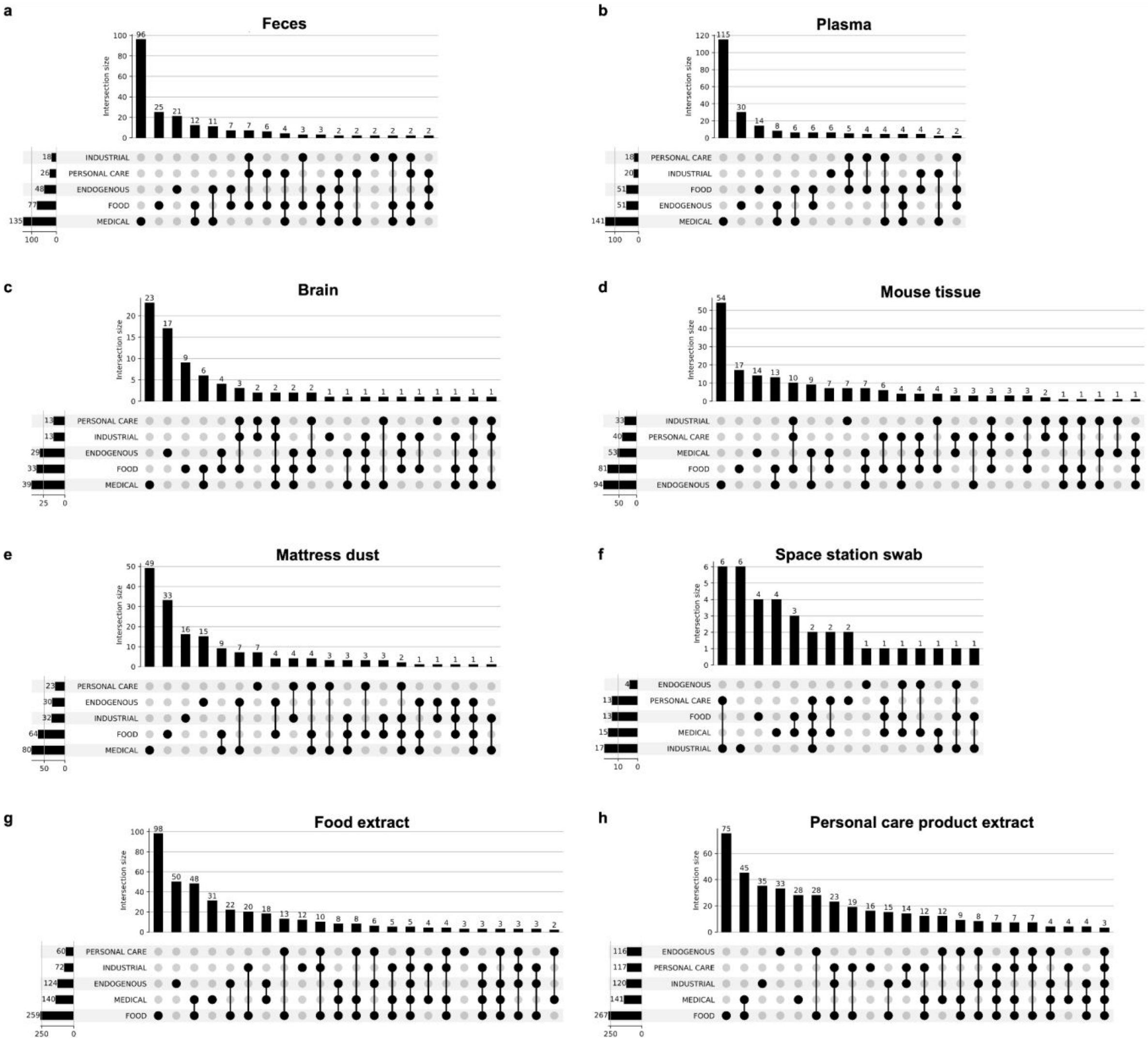

Figure S3. UpSet plots showing the distribution of *chemsource* classification categories for annotated compounds across eight public untargeted metabolomics datasets. These datasets span a diverse range of sample types, including human feces (a), plasma (b), and brain tissue (c) from Alzheimer's disease cohorts; multiple mouse organs (d); dust collected from home mattresses (e); surface swabs from the International Space Station (f); and solvent extracts of food (g) and personal care products (h).

Reference:

(1) PubChem Documentation - *Compounds*. https://pubchem.ncbi.nlm.nih.gov/docs/compounds (accessed 2026-01-09).

(2) Brungs, C.; Schmid, R.; Heuckeroth, S.; Mazumdar, A.; Drexler, M.; Šácha, P.; Dorrestein, P. C.; Petras, D.; Nothias, L.-F.; Veverka, V.; Nencka, R.; Kameník, Z.; Pluskal, T. MSnLib: Efficient Generation of Open Multi-Stage Fragmentation Mass Spectral Libraries. *Nat. Methods* **2025**, *22* (10), 2028–2031.

(3) Zhao, H. N.; Kvitne, K. E.; Brungs, C.; Mohan, S.; Charron-Lamoureux, V.; Bittremieux, W.; Tang, R.; Schmid, R.; Lamichhane, S.; Xing, S.; El Abiead, Y.; Andalibi, M. S.; Mannochio-Russo, H.; Ambre, M.; Avalon, N. E.; Bryant, M.; Burnett, L. A.; Caraballo-Rodríguez, A. M.; Maya, M. C.; Chin, L.; Corominas, L.; Ellis, R. J.; Franklin, D.; Girod, S.; Gomes, P. W. P.; Hansen, L.; Heaton, R. K.; Iudicello, J. E.; Jarmusch, A. K.; Khatib, L.; Letendre, S.; Magyari, S.; McDonald, D.; Mohanty, I.; Cumsille, A.; Moore, D. J.; Rajkumar, P.; Ross, D. H.; Sapre, H.; Shahneh, M. R. Z.; Gil-Solsona, R.; Thomas, S. P.; Tribelhorn, C.; Tubb, H. M.; Walker, C.; Wang, C. X.; Zemlin, J.; Zuffa, S.; Wishart, D. S.; Gago-Ferrero, P.; Kaddurah-Daouk, R.; Wang, M.; Raffatellu, M.; Zengler, K.; Pluskal, T.; Xu, L.; Knight, R.; Tsunoda, S. M.; Dorrestein, P. C. A Resource to Empirically Establish Drug Exposure Records Directly from Untargeted Metabolomics Data. *Nat. Commun.* **2025**, *16* (1), 10600.

(4) Dionisio, K. L.; Phillips, K.; Price, P. S.; Grulke, C. M.; Williams, A.; Biryol, D.; Hong, T.; Isaacs, K. K. The Chemical and Products Database, a Resource for Exposure-Relevant Data on Chemicals in Consumer Products. *Sci. Data* **2018**, *5* (1), 180125.

(5) Orešič, M.; Karu, N.; Zhao, H. N.; Moseley, A.; Hankemeier, T.; Wishart, D. S.; Dorrestein, P. C.; Fiehn, O.; Hyötyläinen, T.; Daouk, R. K. Metabolome Informs about the Chemical Exposome and Links to Brain Health. *Environ. Int.* **2025**, *203*, 109741.

(6) Bennett, D. A.; Buchman, A. S.; Boyle, P. A.; Barnes, L. L.; Wilson, R. S.; Schneider, J. A. Religious Orders Study and Rush Memory and Aging Project. *J. Alzheimers Dis. JAD* **2018**, *64* (Suppl 1), S161–S189.

(7) Zhao, H. N.; Thomas, S. P.; Zylka, M. J.; Dorrestein, P. C.; Hu, W. Urine Excretion, Organ Distribution, and Placental Transfer of 6PPD and 6PPD-Quinone in Mice and Potential Developmental Toxicity through Nuclear Receptor Pathways. *Environ. Sci. Technol.* **2023**, *57* (36), 13429–13438.

(8) Salido, R. A.; Zhao, H. N.; McDonald, D.; Mannochio-Russo, H.; Zuffa, S.; Oles, R. E.; Aron, A. T.; Abiead, Y. E.; Farmer, S.; González, A.; Martino, C.; Mohanty, I.; Parker, C. W.; Patel, L.; Gomes, P. W. P.; Schmid, R.; Schwartz, T.; Zhu, J.; Barratt, M. R.; Rubins, K. H.; Chu, H.; Karouia, F.; Venkateswaran, K.; Dorrestein, P. C.; Knight, R. The International Space Station Has a Unique and Extreme Microbial and Chemical Environment Driven by Use Patterns. *Cell* **2025**, *188* (7), 2022-2041.e23.

(9) Wang, M.; Carver, J. J.; Phelan, V. V.; Sanchez, L. M.; Garg, N.; Peng, Y.; Nguyen, D. D.; Watrous, J.; Kapono, C. A.; Luzzatto-Knaan, T.; Porto, C.; Bouslimani, A.; Melnik, A. V.; Meehan, M. J.; Liu, W.-T.; Crüsemann, M.; Boudreau, P. D.; Esquenazi, E.; Sandoval-Calderón, M.; Kersten, R. D.; Pace, L. A.; Quinn, R. A.; Duncan, K. R.; Hsu, C.-C.; Floros, D. J.; Gavilan, R. G.; Kleigrewe, K.; Northen, T.; Dutton, R. J.; Parrot, D.; Carlson, E. E.; Aigle, B.; Michelsen, C. F.; Jelsbak, L.; Sohlenkamp, C.; Pevzner, P.; Edlund, A.; McLean, J.; Piel, J.; Murphy, B. T.; Gerwick, L.; Liaw, C.-C.; Yang, Y.-L.; Humpf, H.-U.; Maansson, M.; Keyzers, R. A.; Sims, A. C.; Johnson, A. R.; Sidebottom, A. M.; Sedio, B.

E.; Klitgaard, A.; Larson, C. B.; P, C. A. B.; Torres-Mendoza, D.; Gonzalez, D. J.; Silva, D. B.; Marques, L. M.; Demarque, D. P.; Pociute, E.; O'Neill, E. C.; Briand, E.; Helfrich, E. J. N.; Granatosky, E. A.; Glukhov, E.; Ryffel, F.; Houson, H.; Mohimani, H.; Kharbush, J. J.; Zeng, Y.; Vorholt, J. A.; Kurita, K. L.; Charusanti, P.; McPhail, K. L.; Nielsen, K. F.; Vuong, L.; Elfeki, M.; Traxler, M. F.; Engene, N.; Koyama, N.; Vining, O. B.; Baric, R.; Silva, R. R.; Mascuch, S. J.; Tomasi, S.; Jenkins, S.; Macherla, V.; Hoffman, T.; Agarwal, V.; Williams, P. G.; Dai, J.; Neupane, R.; Gurr, J.; Rodríguez, A. M. C.; Lamsa, A.; Zhang, C.; Dorrestein, K.; Duggan, B. M.; Almaliti, J.; Allard, P.-M.; Phapale, P.; Nothias, L.-F.; Alexandrov, T.; Litaudon, M.; Wolfender, J.-L.; Kyle, J. E.; Metz, T. O.; Peryea, T.; Nguyen, D.-T.; VanLeer, D.; Shinn, P.; Jadhav, A.; Müller, R.; Waters, K. M.; Shi, W.; Liu, X.; Zhang, L.; Knight, R.; Jensen, P. R.; Palsson, B. O.; Pogliano, K.; Linington, R. G.; Gutiérrez, M.; Lopes, N. P.; Gerwick, W. H.; Moore, B. S.; Dorrestein, P. C.; Bandeira, N. Sharing and Community Curation of Mass Spectrometry Data with Global Natural Products Social Molecular Networking. *Nat. Biotechnol.* **2016**, *34* (8), 828–837.

(10) Schmid, R.; Heuckeroth, S.; Korf, A.; Smirnov, A.; Myers, O.; Dyrlund, T. S.; Bushuiev, R.; Murray, K. J.; Hoffmann, N.; Lu, M.; Sarvepalli, A.; Zhang, Z.; Fleischauer, M.; Dührkop, K.; Wesner, M.; Hoogstra, S. J.; Rudt, E.; Mokshyna, O.; Brungs, C.; Ponomarov, K.; Mutabdžija, L.; Damiani, T.; Pudney, C. J.; Earll, M.; Helmer, P. O.; Fallon, T. R.; Schulze, T.; Rivas-Ubach, A.; Bilbao, A.; Richter, H.; Nothias, L.-F.; Wang, M.; Orešič, M.; Weng, J.-K.; Böcker, S.; Jeibmann, A.; Hayen, H.; Karst, U.; Dorrestein, P. C.; Petras, D.; Du, X.; Pluskal, T. Integrative Analysis of Multimodal Mass Spectrometry Data in MZmine 3. *Nat. Biotechnol.* **2023**, 1–3.

(11) Nothias, L.-F.; Petras, D.; Schmid, R.; Dührkop, K.; Rainer, J.; Sarvepalli, A.; Protsyuk, I.; Ernst, M.; Tsugawa, H.; Fleischauer, M.; Aicheler, F.; Aksenov, A. A.; Alka, O.; Allard, P.-M.; Barsch, A.; Cachet, X.; Caraballo-Rodriguez, A. M.; Da Silva, R. R.; Dang, T.; Garg, N.; Gauglitz, J. M.; Gurevich, A.; Isaac, G.; Jarmusch, A. K.; Kameník, Z.; Kang, K. B.; Kessler, N.; Koester, I.; Korf, A.; Le Gouellec, A.; Ludwig, M.; Martin H., C.; McCall, L.-I.; McSayles, J.; Meyer, S. W.; Mohimani, H.; Morsy, M.; Moyne, O.; Neumann, S.; Neuweger, H.; Nguyen, N. H.; Nothias-Esposito, M.; Paolini, J.; Phelan, V. V.; Pluskal, T.; Quinn, R. A.; Rogers, S.; Shrestha, B.; Tripathi, A.; van der Hooft, J. J. J.; Vargas, F.; Weldon, K. C.; Witting, M.; Yang, H.; Zhang, Z.; Zubeil, F.; Kohlbacher, O.; Böcker, S.; Alexandrov, T.; Bandeira, N.; Wang, M.; Dorrestein, P. C. Feature-Based Molecular Networking in the GNPS Analysis Environment. *Nat. Methods* **2020**, *17* (9), 905–908.
